# Lives saved from malaria prevention in Africa--evidence to sustain cost-effective gains

**DOI:** 10.1186/1475-2875-11-94

**Published:** 2012-03-28

**Authors:** Eline L Korenromp

**Affiliations:** 1 Global Fund to Fight AIDS, Tuberculosis and Malaria, Vernier, Geneva CH-1214, Switzerland; 2 Department of Public Health, Erasmus MC, University Medical Center Rotterdam, Rotterdam, Netherlands

**Keywords:** Malaria/economics, Malaria/prevention and control, Mortality, Child, Programme impact, Programme evaluation, Financing, Health resources, Investments, Africa

## Abstract

Lives saved have become a standard metric to express health benefits across interventions and diseases. Recent estimates of malaria-attributable under-five deaths prevented using the Lives Saved tool (LiST), extrapolating effectiveness estimates from community-randomized trials of scale-up of insecticide-treated nets (ITNs) in the 1990s, confirm the substantial impact and good cost-effectiveness that ITNs have achieved in high-endemic sub-Saharan Africa. An even higher cost-effectiveness would likely have been found if the modelling had included the additional indirect mortality impact of ITNs on preventing deaths from other common child illnesses, to which malaria contributes as a risk factor.

As conventional ITNs are being replaced by long-lasting insecticidal nets and scale-up is expanded to target universal coverage for full, all-age populations at risk, enhanced transmission reduction may--above certain thresholds--enhance the mortality impact beyond that observed in the trials of the 1990s. On the other hand, lives saved by ITNs might fall if improved malaria case management with artemisinin-based combination therapy averts the deaths that ITNs would otherwise prevent.

Validation and updating of LiST's simple assumption of a universal, fixed coverage-to-mortality-reduction ratio will require enhanced national programme and impact monitoring and evaluation. Key indicators for time trend analysis include malaria-related mortality from population-based surveys and vital registration, vector control and treatment coverage from surveys, and parasitologically-confirmed malaria cases and deaths recorded in health facilities. Indispensable is triangulation with dynamic transmission models, fitted to long-term trend data on vector, parasite and human populations over successive phases of malaria control and elimination.

Sound, locally optimized budget allocation including on monitoring and evaluation priorities will benefit much if policy makers and programme planners use planning tools such as LiST - even when predictions are less certain than often understood. The ultimate success of LiST for supporting malaria prevention may be to prove its linear predictions less and less relevant.

## Background

Ongoing scale-up of malaria control in an era of leveling funds for health requires that investments are justified in terms of their impact and value for money [1]. The Roll Back Malaria partnership, United Nations (through the Millennium Development Goals) and World Health Assembly share a target of reducing malaria deaths by 75% between 2000 and 2015; updated Roll Back Malaria targets of May 2011 include an even more ambitious reduction to near-zero malaria deaths by 2015 [2]. Achieving these targets depends critically on the success of malaria prevention, in particular the provision of insecticide-treated nets (ITNs) in sub-Saharan Africa, the region covering 91% of global malaria deaths. Encouragingly, from 2000 to 2010 nine (out of 43) malaria-endemic African countries, as well as 35 (out of 53) Asian countries with ongoing transmission achieved a more than 50% reduction in notified malaria cases [2].

## Discussion

Lives saved have become a standard metric to express these benefits across interventions and diseases [3-8]. The study by Eisele and colleagues [9] was long-awaited to confirm the large child health gains realized by malaria vector control, primarily ITNs. Across 36 African countries, ITN scale-up is estimated to have reduced malaria mortality deaths by 24% in year 2010, and to have prevented 842,800 under-five deaths between 2001 and 2010. These estimates translate into a cost of US$ 2,770 per life saved or US$ 111 per DALY averted, when compared to the counterfactual of malaria mortality levels as in 2000, when ITN coverage was negligible in most countries.

The Lives Saved Tool (a.k.a. LiST) used by Eisele and colleagues is a suitable mortality projection model, that has the advantage of considering the impact of malaria interventions in the context of multiple key child health interventions, and the full envelope of child deaths from all causes [10-12].

The authors qualify their malaria impact estimates as conservative, which is probably correct despite several uncertainties; in any case the presented cost-effectiveness ratios are somewhat below those published earlier [13,14].

First, lives saved were evaluated within an envelope of malaria-attributable deaths, estimated at 17% of all-cause deaths in children 1-59 months in the year 2000 [15]. In the context of estimating child survival gains across multiple disease control programs, this approach avoids the conceptual problem that lives saved across all interventions would add up to more than the total actual deaths in the population. However, in reality the impact of malaria prevention extends beyond direct malaria-attributable deaths, since malaria acts as a risk factor by causing chronic anaemia, malnutrition and generally weakening child health [16,17]. The cluster-randomized ITN trials that provided the basis for ITN efficacy estimates suggest that this indirect effect may be at least as large as the direct effect within malaria-attributable deaths; which is also why all-cause under-five mortality had--rightly--been the primary effectiveness measure in these trials [18].

Second, the ITN trials, reaching a near-universal (80-100%) household possession of ITNs, estimated effectiveness at a stable 17% reduction in all-cause mortality among children 1-59 months, or 5.5 per 1,000 child-years [18]. Re-analysing three of these five trials, Eisele and colleagues translated this effectiveness into a fixed 55% reduction in *malaria-attributable *deaths in the same age group [19]. However, the trial sites differed markedly in baseline malaria transmission intensity (entomological inoculation rates ranging from < 10 to > 300), all-cause under-five mortality levels (13 to 52 per 1,000 child-years) and the proportion of post-neonatal child deaths due to malaria (36-46% in three trials). It is unclear how all three of these effectiveness measures could be constant across the five sites at the same time--and, by extrapolation, some 15 years later across 36 countries, several of which have since markedly reduced under-five mortality and/or malaria burden [10,20-23].

A third uncertainty is how ITN effectiveness plays out at coverage levels different from the near-universal household possession achieved in the trials. Across the 36 countries now analysed, household ownership ranged from 3.5% to 94% in 2010 [9]. LiST assumes simple linearity, but effectiveness in the lowest-coverage countries may be disproportionally lower if dynamic transmission effects would only kick in at high coverage [24]. Conversely: Is a 55% reduction in malaria-attributable mortality, without additional indirect effects on other-cause deaths, a valid maximum effectiveness for universal net coverage? The latter is the more important question looking forward, for two reasons: First, long-lasting insecticidal nets (LLINs) are replacing conventional ITNs that required retreatment to remain effective. Second, since 2007, the WHO recommends personal protection for the full population at risk instead of just children under-five and pregnant women, resulting in increasing numbers of households owning and using not just one but several ITNs [2].

Further re-analysis of data from the 1990s trials will not improve certainty here. More up-to-date reassurance comes from a recent ecological analysis of household surveys across 22 countries. At ITN ownership levels ranging from 1% to 54% (over 2001-2009), ITN household ownership reduced all-cause post-neonatal under-five mortality by 24% (when adjusted for socioeconomic determinants and coverage of other key child health services), more than the 17% in the cluster-randomized trials [25]. While this survey-based analysis could not estimate the corresponding absolute mortality reduction, it suggests that ITN impact might still be fairly stable at 5.5 deaths prevented per 1,000 child-years of protection, despite the secular trend of falling child mortality.

Enhanced validation of ITN/LLIN effectiveness assumptions would require model fitting to individual settings that scaled-up ITN coverage and measured all-cause and malaria-attributable mortality trends, in the absence of change in other child health interventions. This situation is--fortunately--becoming rarer, as countries scale-up vector control alongside improved malaria treatment [26,27], and as part of broader health systems strengthening. A recent 'validation' study by the same authors found that LiST-predictions fitted child mortality reductions within the 95% CI of empirical observations across two of the ITN trials and two observational studies. However, of these individual sites two had a 'fit' 33-35% too high, and two a 'fit' 22-25% too low [28]--suggesting that the malaria community remains far from a good understanding of ITN's protective efficacy and its determinants across sites.

Validation against recent programme scale-up and mortality trends will furthermore help to monitor whether mortality patterns as of 2000 (adjusted only for population growth) remain the best counterfactual. Even if ITN usage stopped altogether, malaria-related mortality may not resurge to levels as high as in 2000, since child deaths from other major--interacting--causes such as measles, pneumonia and HIV have fallen since [29,30]. Also is malaria treatment with ACT, by lowering case fatality rates, reducing the envelope of potentially fatal malaria deaths that ITNs would prevent [24]. Finally, indoor residual spraying (IRS) is compounding the impact of ITNs, although precise estimates of IRS impact at a regional level are still lacking due to missing data on population-level coverage from some countries.

Next ambitions should focus on enhancing mortality measurement, and on better understanding the impact of vector control in settings of varying endemicity, mortality and anti-malarial treatment patterns. This requires better population-based data on the effective coverage of ACT-based treatment [31] and IRS, and of impact indicators such as parasite infection and anaemia in children [32]. As the coverage of parasitological diagnosis (through microscopy and rapid diagnostic tests) is scaled-up, routine statistics on hospitalizations and in-hospital deaths from parasitologically confirmed malaria will also become an increasingly reliable source [2,23,27,33]. For mortality, the long-term goal for all countries should be to achieve complete and accurate vital registration, classifying causes of death with the International Coding of Deaths system version 10 (ICD-10) [34]. Where nation-wide vital registration is not feasible, sample vital registration or demographic surveillance using verbal autopsy to establish causes of death can be a cost-effective *interim *approach [35,36].

Synthesizing new insights from improved impact data requires a very different type of models, simulating interactions between vector, parasite and human populations over successive phases of malaria control and elimination [24,37]. Dynamic transmission modelling, fitted in detail to selected sites, is indispensable to refine assumptions for simplified health sector planning tools like LiST.

Meanwhile, LiST remains useful to guide short-term strategic planning and M&E priorities in endemic countries, notably reinforcing international recommendations to measure child service coverage and impact indicators through nationally representative household surveys. The pending incorporation of ACT-based treatment [31] will improve LiST's value for programme budgeting--by illustrating the efficiencies expected by shifting allocations from presumptive (symptom-based) treatment that inevitably wastes--scarce--ACT courses on patients with non-malarial fevers, to increased diagnosis and prevention.

## Conclusions

Malaria elimination is becoming a tempting goal even for several currently endemic African countries. But recent gains remain fragile in view of evolving drug and insecticide resistance, malaria's epidemic nature, and the challenge to sustain programme funding despite many competing development priorities that are all (very) cost-effective [2]. Sound, locally optimized budget allocations will benefit much if policy makers and programme planners use simple tools as LiST--even when predictions are much less certain than often understood. The ultimate success of LiST for supporting malaria prevention may be to prove its linear predictions less and less relevant.

## Disclaimer

My views do not necessarily represent the decisions, policy or views of the Global Fund to Fight AIDS, Tuberculosis and Malaria. I declare that I have no conflicts of interest.
